# Multiple Biomarker Simultaneous Detection in Serum via a Nanomaterial-Functionalized Biosensor for Ovarian Tumor/Cancer Diagnosis

**DOI:** 10.3390/mi13122046

**Published:** 2022-11-22

**Authors:** Yu Wu, Chunhua Wang, Chao Wang, Pan Wang, Xiaohan Chang, Lin Han, Yu Zhang

**Affiliations:** 1 Peking University Third Hospital, Haidian District, Beijing 100191, China; 2 Institute of Marine Science and Technology, Shandong University, Qingdao 266273, China; 3 Shenzhen Research Institute of Shandong University, Shenzhen 518057, China; 4 Shandong Engineering Research Center of Biomarker and Artificial Intelligence Application, Jinan 250100, China

**Keywords:** graphene oxide, fluorescence biosensor, protein biomarker, microfluidic, ovarian cancer

## Abstract

Ovarian tumors/cancers are threatening women’s health worldwide, which demands high-performance detection methods and accurate strategies to effectively detect, diagnose and treat them. Here, we report a nanographene oxide particle-functionalized microfluidic fluorescence biosensor to simultaneously detect four biomarkers, CA125, HE4, CEA and APF, for ovarian tumor/cancer diagnosis. The developed biosensor exhibits good selectivity and a large biomarker detection range with a limit of detection of 0.01 U/mL for CA125 and ~1 pg/mL for HE4, CEA and APF. The current results indicate that (1) the proposed biosensor is a promising tool for the simultaneous detection of multiple biomarkers in ovarian tumors/cancer and (2) CA125 and HE4 are strong indicators, AFP may be helpful, and CEA is a weak biomarker for ovarian tumor/cancer diagnosis. The proposed biosensor would be a potential tool, and an analytical approach for the simultaneous detection of multiple biomarkers will provide a new strategy for the early screening, diagnosis and treatment of ovarian tumors/cancers, as well as other cancers.

## 1. Introduction

Ovarian cancer is ranked as the fifth leading cause of cancer death in women worldwide and is divided into 12 substages within 4 main stages. A higher 5-year survival rate (more than 90%) of ovarian cancer is demonstrated if the ovarian tumor/cancer is confirmed and treated at the early stage [1]. Currently, 80% of ovarian cancers are found at the late stages due to their vague symptoms that are often dismissed by women as being associated with aging, menopause, and previous pregnancies at the early stage [2,3]. To improve the early detection rate of ovarian tumors/cancers, routine women’s health checks and screening tests (transvaginal ultrasound (TVUS) and the CA-125 blood test) of ovarian cancer should be performed every year [4]. The protein biomarker CA125 in blood has been used as an effective biomarker for the detection, diagnosis and tracking the recurrence of ovarian tumors/cancer for many years. However, the accurate rate of ovarian cancer detection using CA125 is still low, which requires other biomarkers to compensate for the diagnostic weakness of CA125 [5]. Among the proteins in blood, human epididymis protein 4 (HE4), alpha-fetoprotein (AFP) and carcinoembryonic antigen (CEA) are approved serum biomarkers for cancer detection [6]. Human epididymis protein 4 is found at elevated levels in blood [7,8] and other biofluids [9,10] and plays a critical role in ovarian tumorigenesis [11] and detecting recurrence in CA125-negative ovarian cancer patients [12]; a high death rate is observed for patients with high HE4 levels in the blood [13]. Carcinoembryonic antigen is a substance found on the surface of some cells, and a high level of CEA in blood is linked to certain cancers. It has been reported that CEA could be a good predictor for ovarian tumors [14]. Alpha-fetoprotein is a glycoprotein that is produced in early fetal life by the liver and certain tumors. Some studies reported that high levels of AFP in the blood could be a sign of certain cancers [15]. Considering the improved detection accuracy of ovarian tumors/cancers, researchers have examined the evaluation of ovarian cancer by detecting two or more protein biomarkers in blood or urine. T. Zhao and W. Hu reported that the combined detection of CA125 with HE4 can improve the sensitivity and specificity of ovarian cancer diagnosis and has certain clinical significance that can guide treatment planning [16]. L. Zhang et al. found that the overall performance of ROMA and HE4 was higher than that of CA125, and biomarkers should be selected based on different pathological types of ovarian tumors/cancers [17]. J. Guo et al. reported that the combined detection of serum CA125, CA199 and CEA was promising for epithelial ovarian cancer diagnosis with relatively high sensitivity and specificity [18]. Unfortunately, there is still a lack of studies on the simultaneous detection of CA125, HE4, CEA and AFP in serum for the evaluation of ovarian tumors/cancers.

To accurately detect tumor/cancer biomarkers in biofluids, high-performance biosensing systems are needed. Numerous approaches have been developed to detect biomarkers for different purposes, including enzyme-linked immunosorbent assay (ELISA) biochips [19], surface-enhanced Raman spectroscopy (SERS) biosensors [20], electrochemical biosensing [21], nanosensors [22] and electrical biosensing [23]. Enzyme-linked immunosorbent assay and electrochemical biosensing, including electrochemical luminescence biosensing, are normal methods and are widely used to detect protein biomarkers in labs and hospitals, which normally require large sample volumes. Surface-enhanced Raman spectroscopy-based nanosensors and electrical biosensors are still on the way to practical applications, although they can achieve better performances than ELISA in some instances. In addition, to simultaneously detect multiple tumor/cancer biomarkers to enhance the diagnosis probability of cancers, several strategies have been developed, mainly focusing on the combination of electronic biosensors [24], SERS [25] and microfluidic and droplet-based multichannel microfluidic techniques [26]. Nanomaterial-functionalized biosensors integrated with microfluidic chips can also obtain significant achievements in tumor/cancer biomarker detection in biofluids for the early screening, diagnosis and tracking of cancers [22]. Graphene oxide (GO) nanomaterials have been proven to be good materials for biosensors due to their biocompatibility, abundant functional groups and interactions with bioagents.

In this work, we report a nanosized GO-functionalized fluorescence microfluidic biosensor for the simultaneous detection and analysis of CA125, HE4, CEA and AFP to potentially diagnose ovarian tumors/cancers. Graphene oxide nanomaterials were used to functionalize the substrate, which results in several merits, including the following: (1) nanosized GO can be immobilized on the sensor substrate through a simple plasma and APTES treatment; (2) nanosized GO has a large surface area to enhance the biomarker absorption, resulting in high sensing performance; (3) the immobilization of antibodies on the nanosized GO occurs through simple π-π stacking without an extra functionalization process; and (4) a high signal-to-background ratio is observed by taking advantage of the fluorescent quenching effect of GO. In addition, to the best of our knowledge, this is the first study to simultaneously detect CA125, HE4, CEA and AFP for ovarian tumor/cancer diagnosis. The work could provide a new approach for ovarian tumor/cancer diagnosis, and may contribute to the early screening, diagnosis and tracking of tumors/cancers.

## 2. Materials and Methods

### 2.1. Materials

Silicon wafers for PDMS (Dow Corning, USA) microfluidic mold fabrication were purchased from the Chinese company Meixin Electronic Technology Co., Ltd (China). Nanosized GO particles were purchased from XFNANO Materials Tech Co., Ltd. (Nanjing, China). CA125, HE4, AFP and CEA were purchased from R&D Systems and Fitzgerald (USA). APTES and BSA were obtained from Sigma-Aldrich (China). The real samples were collected at the Third Hospital of Peking University.

### 2.2. Biosensor Fabrication and Tumor/Cancer Biomarker Detection

**Figure 1:**
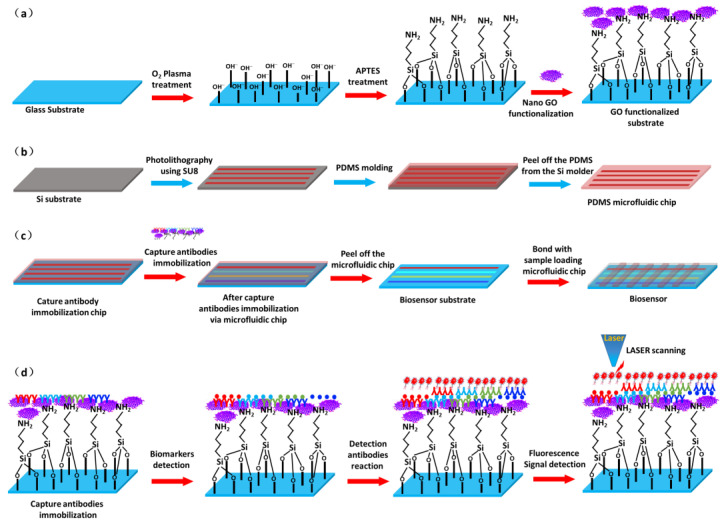
The fabrication schematics of (**a**) the substrate by nanographene oxide functionalization and (**b**) the microfluidic chip. The construction (**c**) and protein biomarker detection (**d**) schematics of the nanographene oxide-functionalized fluorescence microfluidic biosensor.

As shown in Figure 1a, the glass substrates (75 mm × 25 mm) were thoroughly cleaned with piranha solution and then flushed with deionized water, followed by O_2_ plasma treatment for 5 min to produce abundant hydroxyl groups on the surface [27]. Then, the substrates were transferred into an APTES solution. In this step, APTES reacted with hydroxyl groups on the substrate surface, and a thin APTES layer was formed for nanosized graphene oxide immobilization [28]. Finally, the APTES-functionalized substrates were immersed in a 1 mg/mL nanosized GO solution to grow a thin GO layer. The step was completed by rinsing the substrates with DI water 3 times, which were then blown dry using N_2_. The PDMS microfluidic chip fabrication process is shown in Figure 1b. It started with a photolithography process using SU8 to obtain the designed microfluidic chip pattern on the Si substrate. Then, PDMS was poured onto the patterned Si mold and baked. Finally, the PDMS microfluidic chip was separated from the Si mold. More details of the microfluidic chip fabrication can be found elsewhere [29]. It should be noted that there are two types of microfluidic chips. One is the capture antibody immobilization microfluidic chip, which has four channels for the four capture antibodies (CA125, HE4, CEA and AFP). It should be noted that the capture antibodies cannot be immobilized on the substrate without a GO layer. The other is the detection microfluidic chip, in which each channel is aligned on the four capture antibody areas, enabling simultaneous detection of the four biomarkers. To construct the microfluidic fluorescence biosensor, the capture antibody microfluidic chip with 4 channels was bonded with a nanosized GO-functionalized substrate. The CA125, HE4, CEA and AFP capture antibodies at a concentration of 1 mg/mL in 1% BSA solution were immobilized on the substrate through simple π-π stacking between the nanosized GO and antibodies for 30 min to form four parallel antibody lines [30]. Then, the capture antibody microfluidic chip was peeled off in BSA solution. Finally, the detection microfluidic chip was bonded with the antibody-immobilized substrate to form the microfluidic fluorescence biosensor. Figure 1c provides schematics of the antibody immobilization and microfluidic fluorescence biosensor fabrication. To detect ovarian tumor/cancer biomarkers, the samples were loaded into the microfluidic biosensor and reacted with the immobilized capture antibodies for 20 min. Then, allophycocyanin (APC) fluorescence-labeled detection antibodies were loaded and specifically conjugated with the detected antigen for 20 min. The fluorescence-labeled CA125, HE4, CEA and AFP detection antibodies complexed through streptavidin-biotin conjugation were mixed in 1% BSA solution to form detection antibodies. Finally, the detection microfluidic chip was peeled off from the substrate. The substrate was rinsed with PBS and DI water and blown dry with N_2_. The fluorescence signals for distinguishing the different biomarker concentrations in the samples were obtained by scanning the substrate on a GenePix 4400 laser scanner. The detection principle of the protein biomarkers is shown in Figure 1d.

### 2.3. Material and Biosensor Characterizations

The size of the GO was characterized by high-resolution transmission electron microscopy (TEM) (FEI-G20, USA). A smart SPM AFM system was used to characterize the surface morphologies of the samples. A Renishaw inVia Raman microscopy system with a 532 nm laser line was used to validate and analyze the fabrication of the biosensor at room temperature. A GenePix 4400 laser scanner with a resolution of 10 µm and a 635 nm laser source was used to scan the samples to obtain the fluorescence signals, and GenePix software was used to process the scanned fluorescence signals to analyze the results of the samples. There are two reasons to choose the 635 nm channel; one is that the 635 nm channel would not excite GO on the substrate. The other is that the APC-tag on the detection antibody was designed to be excited by a 635 nm laser source.

## 3. Results and Discussion

### 3.1. Characterization of the GO Nanomaterial-Functionalized Biosensor

**Figure 2:**
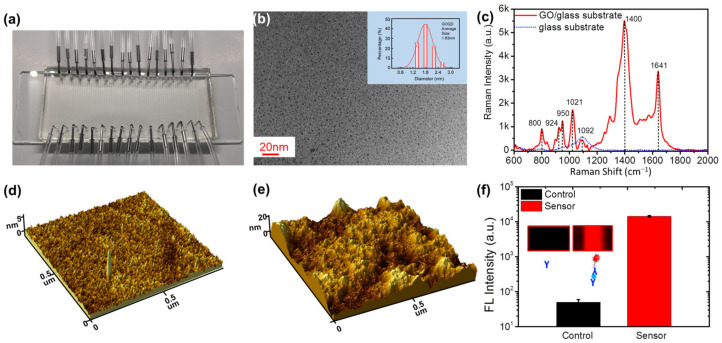
Characterization of the graphene oxide nanoparticle-functionalized fluorescence microfluidic biosensor. (**a**) Optical photograph of the biosensor. (**b**) TEM image of the GO nanoparticles. Inset: Size distribution of GO nanoparticles. (**c**) Raman spectra of the glass substrate and nanographene oxide particle-functionalized substrate. The AFM images of (**d**) the graphene oxide nanoparticle-functionalized substrate and (**e**) capture antibody-immobilized substrate. (**f**) The detected fluorescence intensity with nontarget protein and target protein by the graphene oxide nanoparticle-functionalized fluorescence microfluidic biosensor.

Figure 2 shows the optical photograph of the fabricated microfluidic fluorescence biosensor and the characterizations of the nanomaterial-functionalized biosensor. The mean size of the GO nanoparticles is ~2 nm, as shown in Figure 2b. To validate the GO functionalization of the substrate, Raman spectroscopy was performed on the substrate (Figure 2c). The result clearly shows the GO representative bands at 1401 and 1642 cm^−1^ for the nanosized GO nanomaterials [31], the representative peaks at 800 and 1092 cm^−1^ for the glass [32], and the representative peaks at 924 and 950 cm^−1^ for APTES [33]. The representative Raman peaks of GO, APTES and glass strongly confirmed the successful fabrication of the GO-functionalized substrate. The surface roughness of the GO nanomaterial-functionalized substrate increased from 0.32 to 4.71 nm after the tumor/cancer antibodies were immobilized on the substrate, as shown in Figure 2d,e, indicating the successful immobilization of the antibodies on the substrate. The large surface area of the substrate nanoscale rough surface benefits the high performance of the biosensor resulting from high-density antibody immobilization [34]. Before running detailed biomarker detection, we validated the biosensor by comparing the detected target and nontarget biomarker results on the substrate. As shown in Figure 2f and the inset of Figure 2f, the biosensor shows high fluorescence intensity after the target tumor/cancer antigen and fluorescence-labeled detection antibody reacted with the biosensor, while nontarget biomarker detection exhibited a blank state under the same conditions. All the results confirmed that the GO nanomaterial-functionalized substrate could be a good sensor for biomarker detection in ovarian tumors/cancers.

### 3.2. Sensitivity of CA125, HE4, CEA and AFP Detection

**Figure 3:**
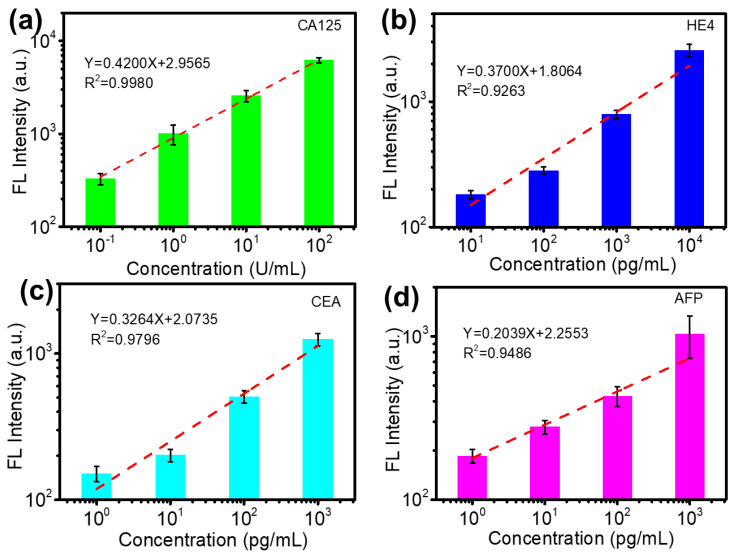
The relationship between detected fluorescence intensity and concentrations of (**a**) CA125, (**b**) HE4, (**c**) CEA and (**d**) AFP in the samples. The dotted lines are the simulated linear curves based on the detected signals; The error bars are from three different tests.

Detection sensitivity is one of the important characteristics of biosensors. To validate the sensitivity of the biosensor for ovarian tumor/cancer biomarkers, 2 μL CA125, HE4, CEA and AFP antigen samples with different concentrations were loaded on the biosensor by using a microfluidic chip and incubated for 20 min, followed by fluorescence-labeled detection antibody loading and incubation for another 20 min. Then, the biosensor was rinsed and dried under N_2_ blowing after peeling off the detection microfluidic PDMS chip, and the substrate was scanned to obtain the fluorescence signals of the biomarkers. As shown in Figure 3, the detected fluorescence intensity increases with elevated biomarker concentration for all four ovarian tumor/cancer biomarkers, and a linear relationship between the prepared biomarker concentrations and detected fluorescence intensity can be simulated. The limit of detection (LOD) of the biosensor for the CA125, HE4, CEA and AFP biomarkers of ovarian tumors/cancers can be calculated by the following equation [35]:

LOD = 3σ/S

where σ is the gradient of the linear regression equation at the low concentration range from 10 to 100 pg/mL and 0.1 to 10 U/mL, and S is the standard deviation of the background. The calculated LODs are ~0.01 U/mL for CA125 and ~1 pg/mL for the HE4, CEA and AFP biomarkers, which are much lower than the cutoff of 35 U/mL for CA125 and 21 pg/mL, 3.5 ng/mL and 20 ng/mL for HE4, CEA and AFP, respectively, indicating that the proposed biosensor would be a good sensing platform to conduct low-concentration ovarian tumor/cancer biomarker detection for early screening and accurate diagnosis. We also compared the developed biosensor sensitivity with other reported results, as listed in Table S1 [23,34,36]. The developed biosensor showed good sensitivity. The high sensitivity and low LOD of the biosensor for the CA125, HE4, CEA and AFP biomarkers can be attributed to high-density antibody immobilization on the nanosized GO material-functionalized substrate.

### 3.3. Selectivity of CA125, HE4, CEA and AFP Detection

**Figure 4:**
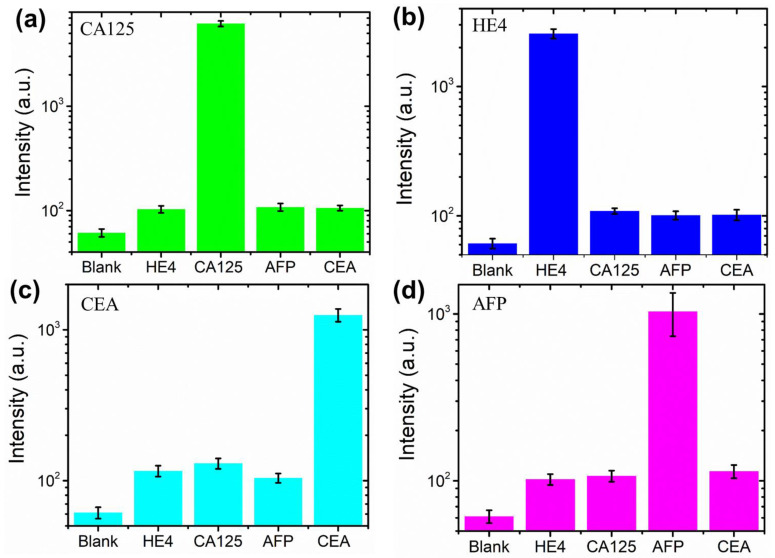
The detection selectivity of (**a**) CA125, (**b**) HE4, (**c**) CEA and (**d**) AFP in the samples. The error bars are from three different tests.

Real samples consist of many biomolecules in addition to the target biomarker, and the biosensor must have a good capability for selective detection of the target biomarker. Therefore, the selectivity of the biosensor was evaluated for CA125, HE4, CEA and AFP biomarkers. Figure 4 shows that the target antigen delivers a much higher fluorescence intensity than other mismatched antigens. The fluorescence intensity of the nontarget proteins was slightly higher than that of the background (blank). The results indicated that all four biomarkers have specific reactions with the target proteins, and the biosensor could specifically detect CA125, HE4, CEA and AFP biomarkers in real samples for ovarian tumor/cancer diagnosis.

### 3.4. Simultaneous Detection of CA125, HE4, CEA and AFP in Stimulated Samples

**Figure 5:**
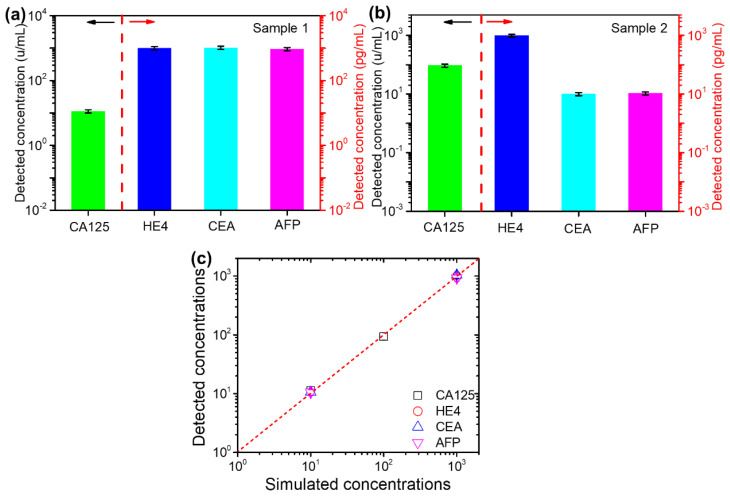
The detection results of the simulated protein biomarker in the serum of simulated sample 1 (**a**) and sample 2 (**b**). The relationship between the simulated and detected sample concentrations (**c**). Note: the dotted line in (**a**,**b**) is the separation line of the Y axis, left side corresponding to left axis, right side corresponding to right axis; the red line in (**c**) is X = Y.

To test the practical application capability of the biosensor, simulated samples in healthy serum with different CA125, HE4, CEA and AFP concentrations were prepared, and the details of the samples are listed in Table 1. The simulated samples are similar to clinical serum because healthy serum contains many complexed biomaterials, which may affect the detection performance of the biosensor. The fluorescence intensity of the biomarkers in the two spiked samples was detected, and the concentrations of the biomarkers were derived based on their relationship equations between the detected fluorescence intensity and concentration, as shown in Figure 3. The detected concentrations are presented in Figure 5a,b. Their recovery varies from 86% to 115%, as shown in Figure 5c, which indicates slight deviation between spiked and detected concentrations for all four biomarkers. The detection results of the simulated samples show that the proposed biosensor is capable of detecting biomarkers in clinical samples.

### 3.5. Detection and Analysis of CA125, HE4, CEA and AFP in Ovarian Tumor/Cancer Real Samples in Our Lab

**Figure 6:**
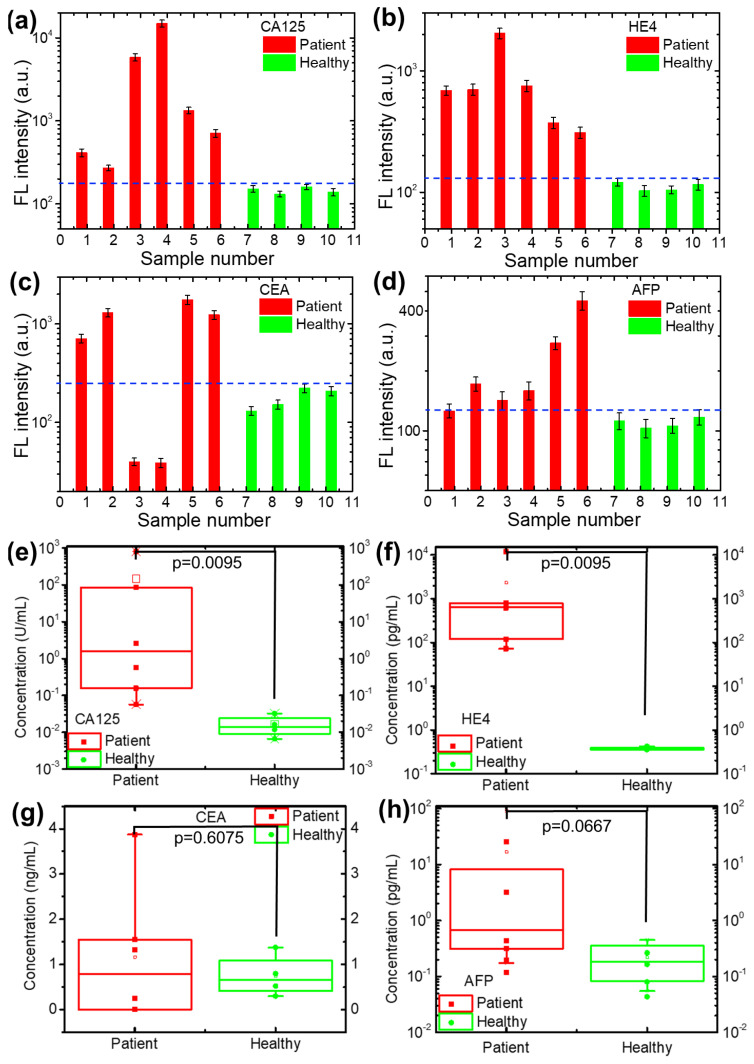
The detected results of six ovarian cancer patients and four healthy people: (**a**) CA125, (**b**) HE4, (**c**) CEA and (**d**) AFP. The blue dotted lines are the highest detection values of the biomarkers from healthy people. The statistical comparison of detected (**e**) CA125, (**f**) HE4, (**g**) CEA and (**h**) AFP in our lab between the ovarian tumor/cancer patients and healthy people.

We applied the developed biosensor to detect and evaluate CA125, HE4, CEA and AFP in real samples from six patients and four healthy people in our laboratory. All the samples were tested using the same biosensor to avoid fluctuations in the detection results due to the biosensor performance. The detected results are presented in Figure 6a–d. The detected CA125, HE4 and AFP biomarker fluorescence intensities of all the ovarian tumor/cancer patients were higher than those of the healthy people, while two of the six ovarian tumor/cancer patients had lower detected fluorescence intensities than the healthy people for the CEA biomarker. To further analyze the difference between patients and healthy people for the four ovarian tumor/cancer biomarkers, a *t* test was applied to the detected results, which is a type of inferential statistic used to determine if there is a significant difference between the means of two groups with data numbers of less than 30. The concentrations of the four biomarkers were derived from the detected fluorescence intensity based on the linear relationship equations shown in Figure 3. The statistical analysis of the four biomarkers is shown in Figure 6e,f. The concentrations of CA125 and HE4 presented statistically significant differences between ovarian tumor/cancer patients and healthy people, with a *p* value of 0.0095 for both CA125 and HE4. The *p* values for CEA and AFP were 0.6075 and 0.0667, respectively, meaning that the concentrations of CEA and APF for ovarian tumor/cancer patients and healthy people were less significantly different. The comparison analysis of the current biomarker test results between patients and healthy people indicated that (1) the proposed biosensor is a promising tool for the simultaneous detection of ovarian tumor/cancer biomarkers and (2) CA125 and HE4 are strong indicators, AFP may be helpful, and CEA is a weak biomarker for ovarian tumor/cancer diagnosis. The results need to be further validated by testing more samples in the future.

## 4. Conclusions

In summary, a GO nanomaterial-functionalized microfluidic fluorescence biosensor fabricated by a simple process is proposed for the simultaneous detection of CA125, HE4, APF and CEA in serum for ovarian tumor/cancer screening and diagnosis. The biosensor delivered good performance with good selectivity, low LOD and a large detection range with high sensitivity for the simultaneous detection of multiple ovarian tumor/cancer biomarkers. The test results and comparison of the four biomarkers in the ovarian tumor/cancer patients and healthy people confirm the capability of the proposed biosensor for practical application in the diagnosis of ovarian tumors/cancer. The current results indicate that CA125 and HE4 are strong indicators, AFP may be helpful, and CEA is a weak biomarker for ovarian tumor/cancer diagnosis, and more samples need to be tested to further validate the results in the future. This work would provide a practical tool for the simultaneous detection of multiple biomarkers and a new strategy for the early screening and diagnosis of ovarian cancer, as well as other cancers.

## Figures and Tables

**Table 1:** Concentrations of the biomarkers spiked in healthy serum.

Sample	CA125 (u/mL)	HE4 (pg/mL)	CEA (pg/mL)	AFP (pg/mL)
1	10	1000	1000	1000
2	100	1000	10	10

## Data Availability

Not applicable.

## Supplementary Materials

The following supporting information can be downloaded at: https://www.mdpi.com/article/10.3390/mi13122046/s1, Table S1: Comparison of sensitivity of different detection methods.
