# Supplementary material for: Multiple Biomarker Simultaneous Detection in Serum via a Nanomaterial-Functionalized Biosensor for Ovarian Tumor/Cancer Diagnosis
Source: Micromachines (Basel). 2022 Nov 22;13(12):2046. doi: 10.3390/mi13122046 (PMC9783278; doi:10.3390/mi13122046)
Supplement: Supplementary file 1 [file micromachines-13-02046-s001.zip › micromachines-2037832-supplementary.pdf]

Table S1. Comparison of sensitivity of different detection methods.

Detection substrate	Detection method	Detection target	Detection limit	Reference
Graphene oxide quantum dots	Elisa	Antigen (CEA, CA125, AFP, CA199, CA153)	~1 pg/mL or 0.01 U/mL	[34]
Graphene	Electrochemical impedence spectroscopy	Antigen (CA125)	~1 ng/μL	[23]
Poly-L-lysine	Elisa	Antigen (IFN-γ, IL-6, TNF-α, etc.)	~10 pg/mL	[36]
Graphene oxide	Elisa	Antigen (CA125, HE4, CEA, AFP)	~1 pg/mL or 0.01 U/mL	This work

References

34. Wang, C.; Zhang, Y.; Tang, W.; Wang, C.; Han, Y.; Qiang, L.; Gao, J.; Liu, H.; Han, L. Ultrasensitive, high-throughput and multiple cancer biomarkers simultaneous detection in serum based on graphene oxide quantum dots integrated microfluidic biosensing platform. *Anal. Chim. Acta.* **2021**, *1178*, 338791, doi:10.1016/j.aca.2021.338791.

23. Gazze, A.; Ademefun, R.; Conlan, R.S.; Teixeira, S.R. Electrochemical impedence spectroscopy enabled CA125 detection; toward early ovarian cancer diagnosis using graphene biosensors. *J. Interdiscip. Nanomed.* **2018**, *3*, 82-88, doi:https://doi.org/10.1002/jin2.40.

36. Lu, Y.; Chen, J.J.; Mu, L.; Xue, Q.; Wu, Y.; Wu, P.H.; Li, J.; Vortmeyer, A.O.; Miller-Jensen, K.; Wirtz, D.; et al. High-throughput secretomic analysis of single cells to assess functional cellular heterogeneity. *Anal. Chem.* **2013**, *85*, 2548-2556, doi:10.1021/ac400082e.
